# DNA methylation links prenatal smoking exposure to later life health outcomes in offspring

**DOI:** 10.1186/s13148-019-0683-4

**Published:** 2019-07-01

**Authors:** Petri Wiklund, Ville Karhunen, Rebecca C. Richmond, Priyanka Parmar, Alina Rodriguez, Maneka De Silva, Matthias Wielscher, Faisal I. Rezwan, Tom G. Richardson, Juha Veijola, Karl-Heinz Herzig, John W. Holloway, Caroline L. Relton, Sylvain Sebert, Marjo-Riitta Järvelin

**Affiliations:** 1 Center for Life Course Health Research, University of Oulu, Oulu, Finland; 2 Department of Epidemiology and Biostatistics, Imperial College London, London, UK; 3 Department of Health Sciences, University of Jyvaskyla, Jyvaskyla, Finland; 4 MRC Integrative Epidemiology Unit, University of Bristol, Bristol, UK; 5 School of Psychology, University of Lincoln, Lincoln, UK; 6 Human Development and Health, Faculty of Medicine, University of Southampton, Southampton, UK; 7 Medical Research Center Oulu, Oulu, Finland; 8 Oulu University Hospital, Oulu, Finland; 9 Research Unit of Clinical Neuroscience, University of Oulu, Oulu, Finland; 10 Institute of Biomedicine and Biocenter of Oulu, Oulu, Finland; 11 Department of Gastroenterology and Metabolism, Poznan University of Medical Sciences, Poznan, Poland; 12 Clinical and Experimental Sciences, Faculty of Medicine, University of Southampton, Southampton, UK; 13 Department for Genomics of Common Diseases, School of Medicine, Imperial College London, London, UK; 14 MRC-PHE Centre for Environment and Health, Imperial College London, London, W2 1PG UK; 15 Department of Life Sciences, College of Health and Life Sciences, Brunel University London, London, UK

**Keywords:** Maternal smoking, Pregnancy, DNA methylation, Persistence, Mediation, Disease, Causality, Life course

## Abstract

**Background:**

Maternal smoking during pregnancy is associated with adverse offspring health outcomes across their life course. We hypothesize that DNA methylation is a potential mediator of this relationship.

**Methods:**

We examined the association of prenatal maternal smoking with offspring blood DNA methylation in 2821 individuals (age 16 to 48 years) from five prospective birth cohort studies and perform Mendelian randomization and mediation analyses to assess whether methylation markers have causal effects on disease outcomes in the offspring.

**Results:**

We identify 69 differentially methylated CpGs in 36 genomic regions (*P* value < 1 × 10^−7^) associated with exposure to maternal smoking in adolescents and adults. Mendelian randomization analyses provided evidence for a causal role of four maternal smoking-related CpG sites on an increased risk of inflammatory bowel disease or schizophrenia. Further mediation analyses showed some evidence of cg25189904 in *GNG12* gene mediating the effect of exposure to maternal smoking on schizophrenia-related outcomes.

**Conclusions:**

DNA methylation may represent a biological mechanism through which maternal smoking is associated with increased risk of psychiatric morbidity in the exposed offspring.

**Electronic supplementary material:**

The online version of this article (10.1186/s13148-019-0683-4) contains supplementary material, which is available to authorized users.

## Background

Maternal smoking during pregnancy is associated with increased risk for pre-term birth, fetal growth restriction, and low birth weight [1–3], as well as neurodevelopmental impairments and respiratory and cardiovascular diseases later in life [4–8]. Despite these well-known risks, many women who commence pregnancy as smokers continue to smoke throughout gestation. According to a recent meta-analysis, the global prevalence of maternal smoking during pregnancy varies widely from a few percentages up to nearly 40% in Ireland [9]. Thus, cigarette smoking continues to be one of the most important modifiable risk factors for the health of mothers and their children.

Cigarette smoke is a potent environmental modifier of DNA methylation [10]. In support of this, an epigenome-wide meta-analysis of 13 birth cohort studies identified over 6000 differentially methylated CpGs in the cord blood of newborns exposed to prenatal smoking [11]. Several smaller studies have suggested that some of these methylation changes may persist across childhood and adolescence into adulthood [12–15]. However, questions remain concerning whether such DNA methylation changes endure across the life course and whether they play a mediating role in linking prenatal smoke exposure to later life health outcomes.

**Fig. 1:**
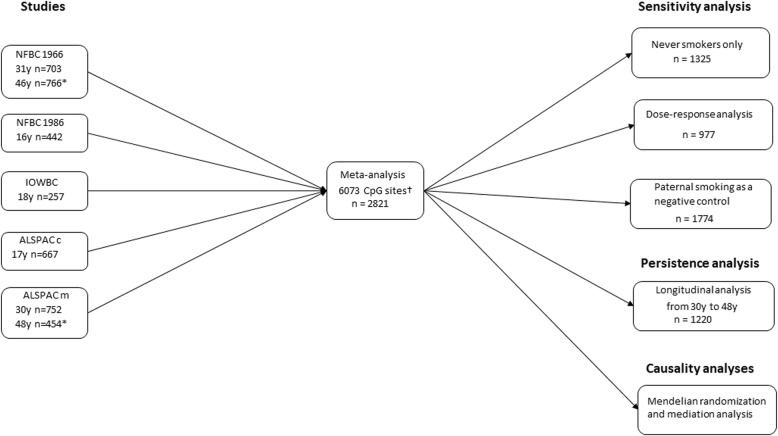
Study design and analytical flow of the study. NFBC Northern Finland Birth Cohort, ALSPAC Avon Longitudinal Study of Parents and Children (m = mothers, c = children), IWBC Isle of Wight Birth Cohort. Dagger symbol denotes CpG sites identified previously in the cord blood of newborns exposed to maternal smoking in utero [11]. Asterisk denotes methylation data for persistence analysis

Here, we combine data from five prospective birth cohort studies to investigate associations between prenatal smoking exposure and offspring blood DNA methylation in 2821 adolescents and adults. We first examine the associations of prenatal smoking exposure with DNA methylation in each cohort and then meta-analyze the results across all studies. We focus on the > 6000 CpG sites previously identified in the cord blood of newborns exposed to prenatal smoking [11]. We further (i) assess the impact of current smoking by the participant on DNA methylation, (ii) explore the dose-dependent effects of prenatal smoking exposure on methylation at key CpG sites, (iii) examine the potential intrauterine effect of smoking exposure on offspring DNA methylation by using paternal smoking as a negative control, (iv) assess the persistence of DNA methylation changes by investigating longitudinal associations from 30 to 48 years of age, and (v) conduct Mendelian randomization (MR) and mediation analyses to examine the potential causal effects of DNA methylation changes on disease outcomes in the offspring (Fig. 1). Our results show that prenatal smoking has persistent effects on the offspring epigenome and provide evidence for a causal role of DNA methylation in adverse health effects that may arise from exposure to tobacco smoke in utero.

## Results

### Cohort-specific characteristics of the study participants

We analyzed the association of prenatal smoking exposure with blood DNA methylation in altogether 1366 adolescents (age 16 to 18 years) and 1455 adults (age 30 to 31 years). Of these, 1145 were from two independent Northern Finland Birth cohorts (NFBC1966 and NFBC1986), 257 were from the Isle of Wight Birth Cohort (IOWBC), and 1419 from two Avon Longitudinal Study of Parents and Children cohorts (ALSPAC mothers and ALSPAC children). Additional file 1, Additional file 2, and Additional file 3 show the characteristics of each study cohort. Overall, 18.4% of the NFBC 1966 and 13.2% of the NFBC 1986 were prenatally exposed to maternal smoking. The corresponding figures were 11.8% for ALSPAC children, 28.7% for ALSPAC mothers, and 16.3% for IOWBC.

### DNA methylation meta-analysis

We found evidence for 69 differentially methylated CpGs in 36 genomic regions (Table 1). All of these CpG sites showed directionally concordant effects with previously reported associations in newborns [11], e.g., hypermethylation of cg04180046 in *MYOG1* and cg05549655 in *CYP1A1* and hypomethylation of cg05575921 in *AHRR* and cg14179389 in *GFI1* in the exposed offspring compared with their unexposed counterparts.

**Table 1:** Association of exposure to maternal smoking during pregnancy and offspring peripheral blood DNA methylation

CpG	Chr	Position	Gene	*β* (SE)	*P* value
cg04180046	7	45002736	*MYO1G*	0.045 (0.003)	2.60E−54
cg12803068	7	45002919	*MYO1G*	0.077 (0.005)	5.70E−45
cg22132788	7	45002486	*MYO1G*	0.045 (0.003)	6.80E−38
cg19089201	7	45002287	*MYO1G*	0.035 (0.003)	1.20E−31
cg05549655	15	75019143	*CYP1A1*	0.010 (0.001)	1.00E−28
cg25949550	7	145814306	*CNTNAP2*	− 0.007 (0.001)	5.40E−27
cg18493761	11	125386885		0.037 (0.004)	7.30E−21
cg15507334	10	14372913	*FRMD4A*	0.024 (0.003)	1.80E−20
cg14179389	1	92947961	*GFI1*	− 0.028 (0.003)	5.00E−20
cg00253658	16	54210496		0.037 (0.004)	3.40E−19
cg17924476	5	323794	*AHRR*	0.024 (0.003)	4.40E−19
cg13570656	15	75019196	*CYP1A1*	0.036 (0.004)	7.40E−19
cg11813497	10	14372879	*FRMD4A*	0.026 (0.003)	9.30E−19
cg22549041	15	75019251	*CYP1A1*	0.041 (0.005)	2.60E−18
cg05575921	5	373378	*AHRR*	− 0.019 (0.002)	3.50E−18
cg12101586	15	75019203	*CYP1A1*	0.032 (0.004)	1.20E−17
cg18092474	15	75019302	*CYP1A1*	0.036 (0.004)	1.50E−17
cg11924019	15	75019283	*CYP1A1*	0.013 (0.002)	2.90E−16
cg25464840	10	14372910	*FRMD4A*	0.019 (0.002)	9.30E−16
cg00213123	15	75019070	*CYP1A1*	0.012 (0.002)	7.80E−15
cg11207515	7	146904205	*CNTNAP2*	− 0.023 (0.003)	1.00E−13
cg14157435	2	206628692	*NRP2*	− 0.046 (0.006)	1.10E−13
cg05348875	2	206628625	*NRP2*	− 0.040 (0.006)	7.60E−13
cg01952185	5	134813213		0.019 (0.003)	3.30E−12
cg22308949	2	206628553	*NRP2*	− 0.022 (0.003)	4.40E−12
cg05204104	2	235403141	*ARL4C*	0.017 (0.003)	4.80E−12
cg11641006	2	235213874		0.017 (0.003)	7.90E−12
cg09935388	1	92947588	*GFI1*	− 0.029 (0.004)	1.10E−11
cg05697249	11	111789693	*C11orf52*	0.015 (0.002)	1.30E−11
cg21161138	5	399360	*AHRR*	− 0.013 (0.002)	1.50E−11
cg26681628	16	54210550		0.018 (0.003)	1.80E−11
cg11025974	2	152830521	*CACNB4*	0.016 (0.002)	9.10E−11
cg15016771	2	235403218	*ARL4C*	0.008 (0.001)	1.00E−10
cg11429111	5	134813329		0.014 (0.002)	1.30E−10
cg25189904	1	68299493	*GNG12*	− 0.020 (0.003)	1.30E−10
cg06758350	21	36259460	*RUNX1*	0.030 (0.005)	1.70E−10
cg17852385	15	75019188	*CYP1A1*	0.010 (0.002)	2.60E−10
cg01664727	21	36258423	*RUNX1*	0.027 (0.004)	2.60E−10
cg20344448	10	14372431	*FRMD4A*	0.014 (0.002)	2.80E−10
cg00794911	6	166260532		− 0.012 (0.002)	5.20E−10
cg22698744	21	36263808	*RUNX1*	0.023 (0.004)	7.30E−10
cg03142697	21	36258497	*RUNX1*	0.016 (0.003)	8.10E−10
cg14563637	9	98931801		0.016 (0.003)	1.20E−09
cg15091747	21	36262896	*RUNX1*	0.014 (0.002)	1.20E−09
cg12984635	19	44032076	*ETHE1*	0.015 (0.002)	2.20E−09
cg01825213	9	98979965		0.019 (0.003)	2.50E−09
cg12477880	21	36259241	*RUNX1*	0.038 (0.006)	2.80E−09
cg17199018	8	28206278	*ZNF395*	− 0.017 (0.003)	3.90E−09
cg00174179	3	49450293	*RHOA;TCTA*	− 0.006 (0.001)	7.00E−09
cg15578140	7	147718109	*MIR548F3;CNTNAP2*	0.011 (0.002)	7.50E−09
cg14540913	9	132458514	*PRRX2*	0.013 (0.002)	7.60E−09
cg18132363	6	166260572		− 0.019 (0.003)	7.60E−09
cg21253335	5	87835928		0.017 (0.003)	1.30E−08
cg25879142	7	4671391		0.018 (0.003)	1.80E−08
cg11845417	11	111789613	*C11orf52*	0.011 (0.002)	2.40E−08
cg20117519	7	8429907		0.022 (0.004)	2.40E−08
cg05783384	2	218843735		0.021 (0.004)	2.60E−08
cg13822849	9	137999757	*OLFM1*	0.007 (0.001)	2.90E−08
cg16449012	4	17781880	*FAM184B*	0.014 (0.002)	3.10E−08
cg05634495	6	122364658		0.016 (0.003)	3.10E−08
cg08644678	4	17711202	*FAM184B*	0.009 (0.002)	3.10E−08
cg13834112	15	90361639		0.013 (0.002)	3.60E−08
cg06635952	2	70025869	*ANXA4*	0.011 (0.002)	5.50E−08
cg14485097	7	4671479		0.016 (0.003)	7.40E−08
cg04598670	7	68697651		− 0.019 (0.003)	8.30E−08
cg03252786	11	125106056	*PKNOX2*	0.006 (0.001)	8.70E−08
cg04749740	2	65935124		0.015 (0.003)	9.20E−08
cg15325070	1	2792704		0.014 (0.003)	9.20E−08
cg04358214	16	67143304	*C16orf70*	0.022 (0.004)	9.60E−08

The analyses were conducted separately in each participating cohort adjusted for study-specific covariates as necessary, and combined using inverse-variance weighted fixed-effects meta-analysis

*Chr* chromosome, *β* effect size estimate, *SE* standard error

### Sensitivity and downstream analyses

**Fig. 2:**
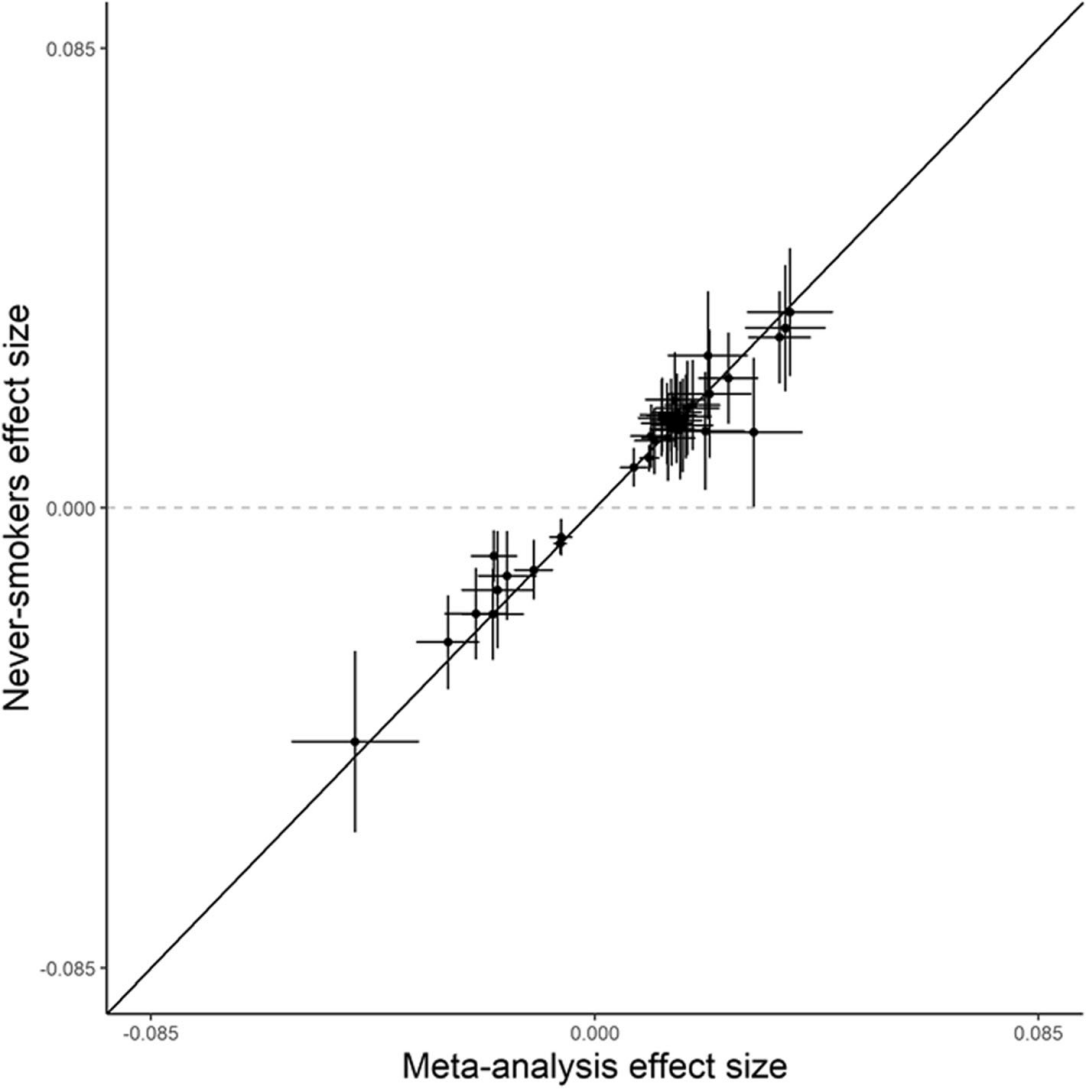
Comparison of meta-analysis effect size estimates and their 95% confidence intervals in all participants (*x*-axis) and never-smokers (*y*-axis) for the 36 top CpG sites. All effect size estimates are adjusted for study-specific covariates as necessary and meta-analyzed using inverse-variance weighted fixed-effects model

To examine whether offspring’s own smoking had influenced the results, we repeated the main analysis including only those individuals who had never smoked regularly in their life. The results were similar, in both direction and magnitude, across all 36 genomic regions as in the full meta-analysis (Fig. 2), indicating that the association between maternal smoking and blood DNA methylation was not mediated through offspring’s own smoking behavior**.**

**Fig. 3:**
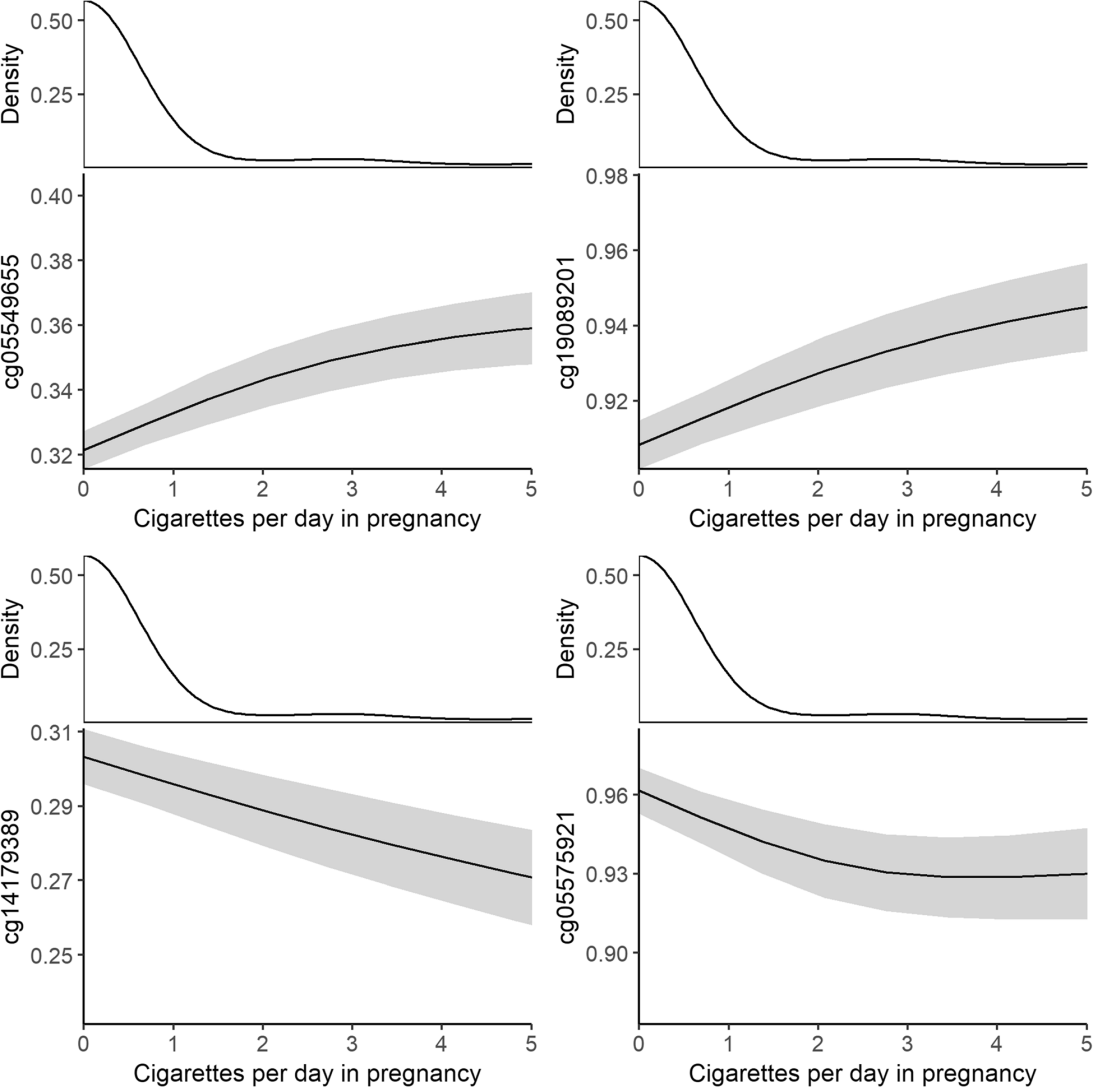
Visualization of the dose-response effect of the intensity of maternal smoking in pregnancy (*x*-axis) on offspring blood DNA methylation (*y*-axis) for top four CpG sites in four gene regions (*AHRR*, *CYP1A1*, *MYO1G*, *GFI1*). Prediction estimates and their 95% confidence intervals plotted based on generalized additive mixed models, with other covariates (offspring sex, body mass index, smoking status, population stratification, and technical covariates) set at their mean (continuous variables) or mode (categorical variables). The density plots represent the distribution of the cigarettes smoked per day in pregnancy. The plots are truncated at five cigarettes per day in pregnancy (containing 94% of full data)

We then examined the dose-response relationship between maternal smoking and blood DNA methylation in the offspring. Methylation differences between the exposed and unexposed offspring became larger with increased smoking intensity across most CpG sites, e.g., each additional three cigarettes smoked per day during pregnancy was associated with 0.23 standard deviation (SD) increase in methylation level in cg05549655 in *CYP1A1* gene (Table 2). Figure 3 shows the visual representations of the dose-response effect of maternal smoking on offspring blood DNA methylation of top CpGs in four top loci.

**Table 2:** Association results for the leading CpG sites from each locus selected for the sensitivity and downstream analyses

			Meta-analysis			Sensitivity and downstream analyses								
			5 studies*, N* = 2821			Never-smokers, *N* = 1298			Paternal smoking, *N* = 1774			Dose-response analysis, *N* = 1134		
CpG	Chr	Gene	*β*	SE	*P* value	*β*	SE	*P* value	*β*	SE	*P* value	*β*	SE	*P* value
cg15325070	1		0.014	0.003	9.23E−08	0.009	0.004	1.92E−02	0.000	0.003	9.16E−01	0.168	0.034	6.81E−07
cg25189904	1	*GNG12*	− 0.020	0.003	1.28E−10	− 0.018	0.004	1.87E−05	− 0.004	0.003	1.52E−01	− 0.146	0.032	5.85E−06
cg14179389	1	*GFI1*	− 0.028	0.003	5.00E−20	− 0.027	0.004	1.07E−09	− 0.004	0.003	1.77E−01	− 0.214	0.031	4.92E−12
cg04749740	2		0.015	0.003	9.21E−08	0.015	0.004	9.64E−04	0.004	0.003	1.03E−01	0.061	0.034	6.98E−02
cg06635952	2	*ANXA4*	0.011	0.002	5.54E−08	0.011	0.003	1.88E−04	0.001	0.002	9.53E−01	0.073	0.029	1.12E−02
cg11025974	2	*CACNB4*	0.016	0.002	9.06E−11	0.018	0.004	1.57E−07	− 0.001	0.002	6.41E−01	0.169	0.033	2.19E−07
cg14157435	2	*NRP2*	− 0.046	0.006	1.15E−13	− 0.042	0.008	6.01E−07	− 0.014	0.006	2.80E−02	− 0.201	0.033	9.33E−10
cg05783384	2		0.021	0.004	2.59E−08	0.014	0.005	1.15E−02	0.003	0.004	3.92E−01	0.097	0.033	3.26E−03
cg05204104	2	*ARL4C*	0.017	0.003	4.81E−12	0.016	0.004	6.02E−05	0.006	0.003	7.96E−02	0.036	0.033	2.73E−01
cg00174179	3	*RHOA*	− 0.006	0.001	7.01E−09	− 0.006	0.002	3.22E−04	− 0.002	0.001	5.21E−02	− 0.104	0.029	3.89E−04
cg16449012	4	*FAM184B*	0.014	0.002	3.10E−08	0.021	0.004	4.42E−08	0.003	0.002	1.62E−01	0.119	0.03	7.81E−05
cg05575921	5	*AHRR*	− 0.019	0.002	3.53E−18	− 0.011	0.003	1.19E−04	− 0.008	0.003	2.75E−03	− 0.134	0.027	5.91E−07
cg21253335	5		0.017	0.003	1.25E−08	0.014	0.004	1.12E−03	0.004	0.003	1.17E−01	0.108	0.033	1.04E−03
cg01952185	5		0.019	0.003	3.31E−12	0.017	0.004	5.81E−05	0.005	0.003	4.49E−02	0.128	0.03	1.67E−05
cg05634495	6		0.016	0.003	3.14E−08	0.014	0.005	2.54E−03	0.002	0.003	5.00E−01	0.088	0.033	6.86E−03
cg00794911	6		− 0.012	0.002	5.20E−10	− 0.007	0.003	1.25E−02	− 0.005	0.002	1.15E−02	− 0.115	0.034	6.42E−04
cg25879142	7		0.018	0.003	1.79E−08	0.013	0.004	3.05E−03	0.003	0.003	3.73E−01	0.121	0.031	1.23E−04
cg20117519	7		0.022	0.004	2.39E−08	0.022	0.006	1.70E−04	0.007	0.004	4.08E−02	0.112	0.033	6.72E−04
cg19089201	7	*MYO1G*	0.035	0.003	1.18E−31	0.030	0.004	5.75E−12	0.007	0.003	1.51E−02	0.225	0.032	2.24E−12
cg04598670	7		− 0.019	0.003	8.33E−08	− 0.016	0.005	4.54E−03	− 0.007	0.003	3.95E−02	− 0.178	0.034	1.54E−07
cg25949550	7	*CNTNAP2*	− 0.007	0.001	5.41E−27	− 0.007	0.001	1.45E−14	− 0.002	0.001	6.57E−03	− 0.157	0.022	1.65E−12
cg11207515	7	*CNTNAP2*	− 0.023	0.003	1.03E−13	− 0.020	0.004	2.75E−06	− 0.011	0.003	2.46E−04	− 0.145	0.032	7.02E−06
cg15578140	7	*MIR548F3*	0.011	0.002	7.54E−09	0.014	0.003	7.89E−06	− 0.001	0.002	5.70E−01	0.152	0.031	1.12E−06
cg17199018	8	*ZNF395*	− 0.017	0.003	3.85E−09	− 0.016	0.004	7.10E−05	− 0.003	0.003	3.10E−01	− 0.121	0.033	2.63E−04
cg14563637	9		0.016	0.003	1.19E−09	0.016	0.004	9.26E−05	0.003	0.002	1.69E−01	0.092	0.031	3.26E−03
cg14540913	9	*PRRX2*	0.013	0.002	7.57E−09	0.015	0.003	1.81E−05	0.001	0.002	5.79E−01	0.148	0.032	2.62E−06
cg13822849	9	*OLFM1*	0.007	0.001	2.87E−08	0.007	0.002	5.84E−05	0.003	0.002	2.90E−02	0.126	0.03	3.24E−05
cg11813497	10	*FRMD4A*	0.026	0.003	9.27E−19	0.022	0.004	1.46E−07	− 0.001	0.003	6.26E−01	0.117	0.032	2.81E−04
cg05697249	11	*C11orf52*	0.015	0.002	1.30E−11	0.015	0.003	8.68E−06	0.003	0.002	1.54E−01	0.136	0.035	8.25E−05
cg18493761	11		0.037	0.004	7.30E−21	0.032	0.006	4.99E−08	0.008	0.004	2.60E−02	0.154	0.034	4.82E−06
cg05549655	15	*CYP1A1*	0.010	0.001	1.04E−28	0.009	0.001	6.03E−13	0.002	0.001	4.28E−02	0.230	0.028	1.92E−16
cg13834112	15		0.013	0.002	3.55E−08	0.018	0.004	6.39E−07	0.001	0.002	8.10E−01	0.120	0.031	1.24E−04
cg00253658	16		0.037	0.004	3.44E−19	0.032	0.006	6.54E−08	0.005	0.005	2.81E−01	0.166	0.033	4.83E−07
cg04358214	16	*C16orf70*	0.022	0.004	9.56E−08	0.021	0.006	5.93E−04	0.005	0.004	2.42E−01	0.090	0.031	3.94E−03
cg12984635	19	*ETHE1*	0.015	0.002	2.17E−09	0.014	0.003	5.87E−05	0.001	0.002	7.33E−01	0.116	0.031	1.49E−04
cg06758350	21	*RUNX1*	0.030	0.005	1.68E−10	0.016	0.007	2.03E−02	0.002	0.005	7.09E−01	0.140	0.034	3.92E−05

The analyses were conducted separately in each participating cohort, adjusted for study-specific covariates as necessary, and combined using inverse-variance weighted fixed-effects meta-analysis. The effect size estimates for the dose-response analysis represent the difference in blood DNA methylation (in standard deviation units) per three additional cigarettes smoked per day in pregnancy

*Chr* chromosome, *β* effect size estimate, *SE* standard error

To assess potential unmeasured confounding and to establish a causal intrauterine effect between maternal smoking and the offspring DNA methylation, we used paternal smoking as a negative control. Maternal smoking and paternal smoking showed similar directions of effect; however, the effect estimates for exposure to paternal smoking were considerably smaller (Table 2). Adjusting for paternal smoking had no significant effect on maternal smoking estimates (Additional file 4).

**Fig. 4:**
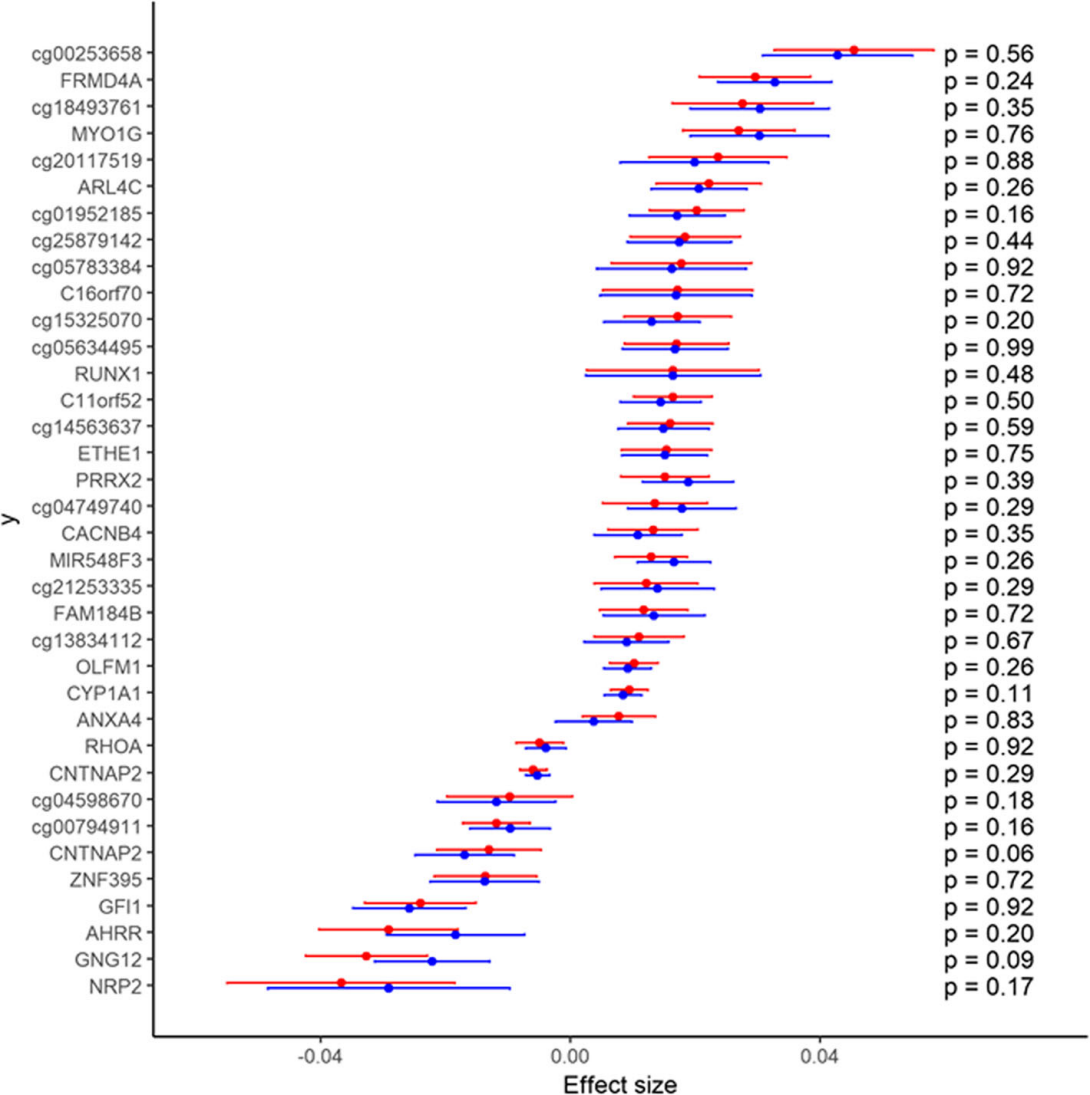
Longitudinal analysis of association between exposure to maternal smoking and offspring blood DNA methylation. Effect size estimates (adjusted for study-specific covariates and meta-analyzed using inverse-variance weighted fixed-effects model) and their 95% confidence intervals at age 30–31 years (red) and age 46–48 years (blue) for top CpG sites and *P* values for the test of equality of the effect size estimates

We performed a longitudinal analysis to examine whether the maternal smoking-associated alterations in DNA methylation persisted from early adulthood (age 30–31 years) into midlife (age 46–48 years) in the NFBC 1966 and ALSPAC mothers’ cohorts. We found no evidence for change in direction or magnitude of associations in blood DNA methylation between the two time points (Fig. 4), suggesting that DNA methylation levels remain relatively stable for several decades after prenatal exposure to maternal smoking.

### Mendelian randomization analysis

We estimated the causal effects of DNA methylation changes on disease outcomes using MR. We extracted the effect sizes of SNP-CpG associations for the 69 differentially methylated CpGs available in the Accessible Resource for Integrated Epigenomic Studies (ARIES) mQTL database [16] (http://www.mqtldb.org/) and found strong instruments for 15 CpG sites. Of these 15 CpG sites, three (cg15578140 in microRNA 548f-3 (*MIR548F3*), cg09935388 in Growth Factor Independent Protein 1 (*GFI1*), cg04598670 (unknown gene)) showed potential causal associations with inflammatory bowel diseases and one (cg25189904 in Guanine Nucleotide Binding Protein Gamma 12 (*GNG12*)) with schizophrenia (*P*_FDR_ < 0.05, Table 3).

**Table 3:** Mendelian randomization analysis of top differentially methylated CpGs tested against 106 diseases

Exposure	Gene	Disease	*β*	SE	*P* value	*FDR P* value	Unit
cg15578140	*MIR548F3*	Inflammatory bowel disease	− 0.104	0.018	3.73E−09	2.54E−07	Log odds
cg09935388	*GFI1*	Inflammatory bowel disease	− 0.152	0.034	7.27E−06	2.18E−04	Log odds
cg04598670	Unknown	Inflammatory bowel disease	− 0.410	0.091	7.27E−06	5.24E−04	Log odds
cg09935388	*GFI1*	Crohn’s disease	− 0.162	0.040	4.74E−05	7.12E−04	Log odds
cg04598670	Unknown	Crohn’s disease	− 0.439	0.108	4.74E−05	1.71E−03	Log odds
cg09935388	*GFI1*	Ulcerative colitis	− 0.160	0.042	1.47E−04	1.47E−03	Log odds
cg04598670	Unknown	Ulcerative colitis	− 0.433	0.114	1.47E−04	3.52E−03	Log odds
cg25189904	*GNG12*	Schizophrenia	− 0.222	0.053	3.37E−05	1.82E−03	Log odds

Only significant associations (*P*_FDR_ < 0.05) are shown

*GFI1* growth factor independent protein 1, *MIR548F3* microRNA 548f-3, *GNG12* guanine nucleotide binding protein gamma 12, *β* effect size estimate, *SE* standard error *FDR* false discovery rate

### Mediation analysis

**Fig. 5:**
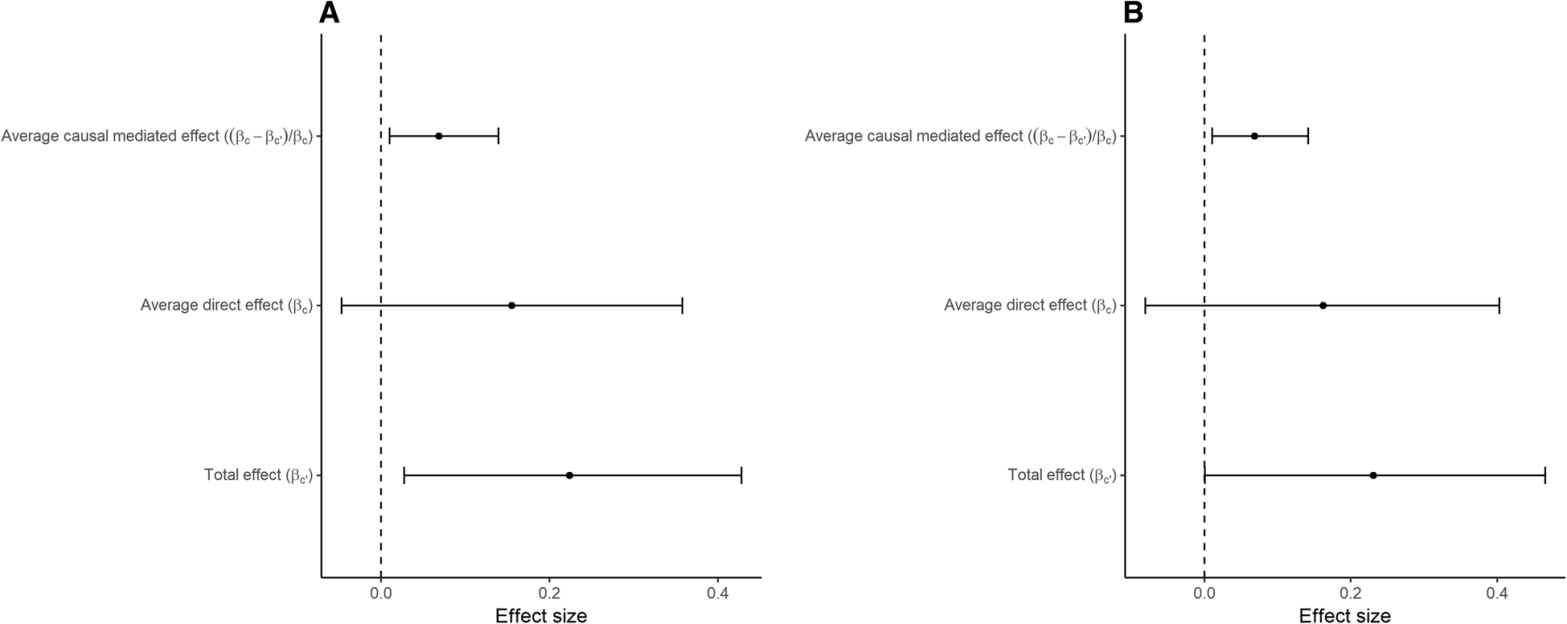
Mediation analysis examining the indirect effect of maternal smoking during pregnancy on Bipolar II Scale (**a**) and Hypomanic personality scale (**b**) through differential methylation of cg25189904 in *GNG12*. Data are shown as beta estimate for effect size and 95% confidence intervals

We then sought to test whether methylation changes in these four CpGs mediated the association between maternal smoking and disease outcomes. However, since the prevalence of inflammatory bowel disease is relatively low in the general population, we assessed the associations of maternal smoking and CpGs on irritable bowel syndrome (IBS), which is a constellation of functional gastrointestinal disorder symptoms. These data were obtained from self-administered questionnaires in NFBC1966 at 46 years [17]. Prevalence of schizophrenia is also low in the general population. Therefore, instead of diagnosed schizophrenia, we used personality trait scales measuring schizotypal and affective symptoms as an outcome. Such personality scales were derived from questionnaires available in the NFBC 1966 data at 31 years, and they can be used to identify subjects with latent personality with genetic vulnerability for schizophrenia [18]. We found evidence for cg25189904 mediating the association between exposure to maternal smoking and Bipolar II Scale (*P* = 0.024) and Hypomanic Personality Scale (*P* = 0.018) (Fig. 5a and b). The estimated mediated proportions were 30% and 28%, respectively (Additional file 5). We did not find evidence for a mediating effect of blood DNA methylation on IBS (*P* > 0.3 for all CpGs, Additional file 5).

## Discussion

We combined data from five studies in adolescents and adults to examine the association between maternal smoking during pregnancy and blood DNA methylation in the offspring from 16 to 48 years of age. We identified 69 differentially methylated CpGs in 36 genomic regions. The top differentially methylated CpG sites showed a clear dose-response relationship with number of cigarettes smoked during pregnancy. The associations observed in adulthood were robust to adjustment for multiple potential confounding factors and persisted into middle age with no significant change in direction and magnitude of associations. Mendelian randomization and mediation analyses suggested that alterations in DNA methylation may link maternal smoking during pregnancy to increased risk of psychiatric morbidity and potentially with inflammatory bowel disease in the exposed offspring.

The findings of our study confirm and extend the results of earlier reports by demonstrating that maternal smoking during pregnancy is associated with alterations in offspring blood DNA methylation not only in newborns [11, 19, 20], children, and adolescents [12, 13], but also in adults, several decades following the exposure. The similarity in differentially methylated CpG sites and the consistency in direction of methylation changes between our study and earlier EWAS imply that the smoke exposure-induced methylation changes may be soma-wide and persist throughout life. However, the effects of smoking may also be targeted to specific regions of the epigenome, as indicated by the observations that both prenatal smoke exposure and active smoking affect the methylation patterns of same gene regions, e.g., *AHRR* and *CYP1A1*, which are involved in chemical detoxification [10]. Because of these similar effects, the methylation changes found in people exposed to prenatal smoking may also reflect current or past smoking by the people themselves or some other passive smoking exposure. Adjusting for offspring active smoking did not substantially change the results in the present study. However, parental smoking is known to associate with their offspring’s smoking behavior also via genetic predisposition [21, 22] and thus own smoking may serve as a mediator on the path between maternal smoking and DNA methylation. Therefore, simply adjusting for own smoking can lead to erroneous conclusions about the direct effects of maternal smoking [23]. We therefore performed a sensitivity analysis including only offspring who themselves had never smoked in their life and found that the associations were similar across all CpG sites as in the full meta-analysis.

We also used paternal smoking as a negative control by comparing the associations of maternal smoking during pregnancy and paternal smoking with offspring methylation and found that the effect estimates were substantially greater for maternal smoking, and adjusting for paternal smoking had virtually no effect on maternal smoking estimates. This indicates it is unlikely that the associations between maternal smoking and offspring methylation were attributable to post-natal passive smoking exposure or some unmeasured confounding. These results together with the finding of a clear dose-dependent relationship of methylation with increased smoking intensity during pregnancy suggest a direct biological effect of in utero exposure to cigarette smoke on DNA methylation.

The longitudinal analysis showed that differentially methylated CpGs observed around age 30 persisted into middle age (around age 48) without significant change in direction or magnitude of methylation levels. This corroborates the findings of recent smaller studies, which found several differently methylated CpGs in middle-aged women exposed to maternal smoking in utero [14, 15], and suggests that some of the prenatal smoking exposure-associated methylation changes are largely irreversible and unaffected by age and/or environmental exposures later in life. To assess whether such persistent changes in DNA methylation are causally implicated with disease, we performed a Mendelian randomization analysis using summary data from large genome-wide association studies [24]. We found evidence for potential causal associations for three CpGs (cg15578140, cg09935388, cg04598670) with inflammatory bowel disease and one CpG (cg25189904) with schizophrenia. To strengthen the evidence for these potentially causal associations, we also performed a formal mediation analysis in the NFBC1966 cohort and found evidence for differential methylation in cg25189904 mediating the association between maternal smoking and Bipolar II Scale and Hypomanic Personality Scale, explaining 30% and 28% of the total effect, respectively. These results corroborate the findings of previous observational studies that maternal smoking during pregnancy is associated with increased risk of psychiatric morbidity in the exposed offspring [25–28]. However, we found no evidence for mediating effect of differential methylation cg15578140, cg09935388, and cg04598670 in the association of maternal smoking and irritable bowel syndrome. Such discrepant results could be due to relatively small sample size in the mediation analysis, or because the irritable bowel syndrome is not a good proxy for inflammatory bowel disease, or because the causal effect estimates for inflammatory bowel disease in the MR analysis were biased due to, for example, pleiotropic effects of genetic instruments on the outcome. Thus, additional studies are needed to assess whether prenatal smoking is associated with increased risk of inflammatory bowel disease in the exposed offspring and whether alterations in DNA methylation mediates this association.

Our results may provide insights into potential mechanisms linking prenatal smoking exposure to psychiatric disorders. Experimental studies suggest that *GNG12* is an important regulator of inflammatory signaling in microglia cells, which are the resident macrophages of the central nervous system [29]. A role of inflammation in the etiology of schizophrenia and psychotic illness has been suggested [30, 31], and in line with this, a large meta-analysis of 2424 cases and over 1.2 million controls indicated that childhood central nervous system infections are associated with nearly twofold risk of schizophrenia in adulthood [32]. Our DNA methylation data were from the whole blood while the pathogenic processes for psychiatric disorders, including schizophrenia, occur primarily in brain tissue. We believe that methylation in blood mirrors the corresponding sites in disease-relevant tissues [33]. Such mirror sites can occur if the exposure occurs during early stages of prenatal development, thus affecting multiple tissues [33]. Therefore, blood DNA methylation may act as a marker for differential DNA methylation in the primary disease tissue that is mediating the effects of intrauterine smoke exposure. There is support justifying the use of blood samples to discover genes related to brain phenotypes and diseases [34]. However, further studies are needed to validate our findings and investigate the biological relevance of *GNG12* in the corresponding tissue.

Our study has both strengths and limitations. The large sample size of males and females and similar ages from different cohorts enabled us to obtain precise estimate of the long-term effects of maternal smoking on DNA methylation. Several downstream analyses and use of paternal smoking as a negative control allowed us to distinguish the associations from potential confounding, and the follow-up analysis from young adulthood to middle age allowed us to examine the persistence of methylation changes. The limitations are that we did not have tissue-specific DNA methylation data as indicated above and that maternal smoking was determined from self-reported questionnaires. As self-reports may be biased by under-reporting or recall bias, our findings may underestimate true effects. In the ALSPAC mothers’ cohort, the adult offspring reported their mothers’ smoking, although this could also be subject to recall bias. False reporting may also concern the adolescents in our study since they might have been reluctant to disclose their true smoking behavior, although in the IOWBC adolescent smoking was confirmed by urinary cotinine measurement. Another limitation is that the subjects in the ALSPAC children and ALSPAC mothers’ cohorts are related individuals. However, excluding either one of the related ALSPAC data sets did not notably affect the results (data not shown).

## Conclusions

Maternal smoking during pregnancy has long-lasting effects on offspring epigenome. DNA methylation may represent a biological mechanism through which maternal smoking is associated with increased risk of psychiatric morbidity and potentially inflammatory bowel disease in the exposed offspring.

## Methods

### Study cohorts

#### Northern Finland Birth Cohort 1966

The Northern Finland Birth Cohort 1966, previously described in detail [35, 36], targeted all pregnant women, residing in the two northernmost provinces of Finland with expected dates of delivery between 1 January and 31 December 1966. Over 96% of eligible women participated in the study, thus comprising 12,055 mothers followed prospectively on average from 16th gestational week and 12,058 live-born children. In 1997, at offspring age of 31 years, all cohort participants with known addresses were sent a postal questionnaire on health and lifestyle and those living in Northern Finland or Helsinki area were invited to a clinical examination which included blood sampling. In total, both questionnaire and clinical data were collected for 6007 participants. DNA was successfully extracted for 5753 participants from fasted blood samples [37]. In 2012, all individuals with known address in Finland were sent postal questionnaires and an invitation for clinical examination. Both questionnaire and clinical data was collected for 5539 participants. DNA methylation at 31 years was extracted for 807 randomly selected subjects of whom both questionnaire and clinical data with cardio-metabolic measures were available at both 31 and 46 years. Of these individuals, DNA methylation data at 46 years was extracted for 766 subjects.

#### Northern Finland Birth Cohort 1986

The Northern Finland Birth Cohort 1986 includes all mothers (prospective data collection from 10th gestational week) with children whose expected date of delivery fell between July 1, 1985, and June 30, 1986, in the two northernmost provinces of Finland (99% of all births during that time) [38]. The cohort consists of 9362 women and 9432 live-born children. In 2001, all individuals with known address received a postal questionnaire on health and lifestyle and invitation to a clinical examination. DNA were extracted from fasting blood samples, and DNA methylation was measured for 546 randomly selected subjects with full data available.

In both NFBC cohorts, complete data included singleton births and subjects with complete set clinical follow-up and DNA methylation data, excluding subjects with missing information and twins. A written informed consent for the use of the data including DNA was obtained from all study participants and their parents. Ethical approval for the study was received from Ethical Committee of Northern Osthrobothnia Hospital District and Oulu University, Faculty of Medicine.

#### Isle of Wight Birth Cohort

Isle of Wight Birth Cohort is a general population-based birth cohort recruited on the Isle of Wight in 1989 to assess the role of heredity and environment on development of allergic disorders and allergen sensitization. The details of this birth cohort have been described in previous reports [39]. In brief, both the Isle of Wight and the study population are 99% Caucasian. Ethics approvals were obtained from the Isle of Wight Local Research Ethics Committee (now named the National Research Ethics Service, NRES Committee South Central—Southampton B) at recruitment and for the 1, 2, 4, 10, and 18 years follow-up. Exact age at 18-year follow-up was calculated from the date of blood sample collection for the 18-year follow-up and the date of birth. DNA methylation in peripheral blood samples was analyzed from randomly selected subjects (*n* = 257) at the 18-year follow-up.

#### Avon Longitudinal Study of Parents and Children

Pregnant women resident in the former county of Avon, UK, with expected dates of delivery 1 April 1991 to 31 December 1992 were invited to take part in the study. The initial number of pregnancies enrolled is 14,541 (for these at least one questionnaire has been returned or a “Children in Focus” clinic had been attended by 19 July 1999). Of these initial pregnancies, there was a total of 14,676 fetuses, resulting in 14,062 live births and 13,988 children who were alive at 1 year of age [40, 41].

The Accessible Resource for Integrated Epigenomic Studies (ARIES) is a sub study of ALSPAC, which includes 1018 mothers and their children for whom methylation data has been created [42]. The ARIES participants were selected based on the availability of DNA samples at two time points for the women (antenatal [mean age 30 years] and at follow-up [mean age 48 years] when the offspring were adolescents) and three time points for their offspring (neonatal, childhood [mean age 7.5 years], and adolescence [mean age 17.1 years]). A web portal allows openly accessible browsing of aggregate ARIES DNA methylation data (ARIES-Explorer) (http://www.ariesepigenomics.org.uk/). Please note that the study website contains details of all the data that is available through a fully searchable data dictionary and variable search tool: http://www.bristol.ac.uk/alspac/researchers/our-data/. Ethical approval for the study was obtained from the ALSPAC Ethics and Law Committee and the Local Research Ethics Committees.

### Definition of maternal smoking during pregnancy

In NFBCs and ALSPAC studies, expectant mothers were asked whether they had smoked cigarettes before or at the beginning of the pregnancy, how many years they had smoked, the number of cigarettes smoked per day, and whether they had changed their smoking habits during the pregnancy. Offspring were considered to be prenatally exposed to cigarette smoking if mother reported smoking regularly (at least one cigarette per day) from pregnancy week 8 onwards. The ALSPAC mothers were also asked whether their mothers had smoked and were asked whether they had smoked when they were pregnant with them. In the IOWBC, maternal smoking status in pregnancy was self-reported and defined as any smoking in pregnancy or no smoking during pregnancy.

### Measurement of DNA methylation

Methylation of genomic DNA was quantified using the Illumina HumanMethylation450 array (ALSPAC, ARIES, IOWBC, and NFBC1966 at age 31, NFBC1986) or Illumina EPIC array (NFBC1966 at age 46) according to the manufacturer’s instructions. Bisulfite conversion of genomic DNA was performed using the EZ DNA methylation kit according to the manufacturer’s instructions (Zymo Research, Orange, CA).

### Quality control of methylation data

In NFBCs and IOWBC, quality control and quantile normalization for DNA methylation data were adapted from the CPACOR pipeline [43]. Illumina Background Correction was applied to the intensity values, a detection *P* value threshold was set at *P* < 10^−16^, and samples with call rate < 98% were excluded. Quantile normalization was done separately for six probe-type categories, and these normalized intensity values were used to calculate the methylation beta value at each CpG site, ranging between 0 (no methylation) and 1 (full methylation). Probes with call rate < 95% were excluded from the analyses. A principal component analysis (PCA) was carried out for array control probes, and the first 30 principal components (PCs) were used as explanatory variables in the subsequent regression models [43]. White blood cell subpopulation estimates were obtained using the software provided by Houseman et al. [44], and these estimates were also added as covariates in the regression models. In ARIES, the DNA methylation wet-laboratory and pre-processing analyses were performed as previously described [42]. In brief, samples from all time points were distributed across slides using a semi-random approach to minimize the possibility of confounding by batch effects. Samples failing quality control (average probe *P* value ≥ 0.01, those with sex or genotype mismatches) were excluded from further analysis and scheduled for repeat assay, and probes that contained < 95% of signals detectable above background signal (detection *P* value < 0.01) were excluded from the analysis. Methylation data were pre-processed using R software, with background correction and subset quantile normalization performed using the pipeline described by Touleimat and Tost [45].

### Statistical analyses

#### Meta-analysis of 6073 CpG sites in five studies

Study design and analytical flow of the study are shown in Fig. 1, and the data availability for each analysis is presented in Table 4. All analyses were conducted using R software [46]. Linear regression was used to examine the association between sustained maternal smoking during pregnancy (from pregnancy week 8 onwards) and offspring peripheral blood DNA methylation at 6073 CpG sites that were previously identified to be differentially methylated in newborns exposed to maternal smoking in utero in recent epigenome-wide association study (EWAS) (false discovery rate-corrected *P* value < 0.05) [11]. The final model was adjusted for study-specific covariates as necessary (offspring’s sex, BMI, smoking status, and social class for IOWBC; additionally first four genetic PCs for NFBC cohorts; offspring age, maternal age, and social class for ALSPAC cohorts). The model was run independently in each study, and the results were then meta-analyzed over all five studies (NFBC1986 (age 16 years), NFBC1966 (age 31 years), IOWBC (age 18 years), ALSPAC mothers (age 30 years) and ALSPAC children (age 17 years)) using an inverse variance weighted fixed-effects model. Statistical significance level was set at *P* < 1 × 10^−7^, which corresponds approximately to a Bonferroni-corrected significance level of 0.05 for 450,000 independent tests. Such a conservative threshold was robust, and thus, the significant probes were considered worthy of further examination in a series of sensitivity and downstream analyses. The leading CpG site from each gene region (1-Mb window centered on the CpG site with the strongest association) was selected for these analyses.

**Table 4:** Data availability in each study for different analyses

			Sensitivity and downstream analyses for top CpG sites			
Study	Mean age at methylation data collection (years)	Association results for cord-blood-methylation associated CpG sites	Never-smokers	Paternal smoking	Amount of cigarettes smoked in pregnancy	Longitudinal methylation data (mean age at the 2nd time point)
NFBC1986	16	Yes	Yes	Yes	Yes	No
NFBC1966	31	Yes	Yes	Yes	Yes	Yes (46 years)
ALSPAC mothers	30	Yes	Yes	No	No	Yes (48 years)
ALSPAC children	17	Yes	Yes	Yes	No	No
IOWBC	18	Yes	Yes	No	No	No

We note that ALSPAC children were part of the earlier study from where the 6073 CpG sites were selected [11]. However, the earlier study examined DNA methylation in the cord blood, whereas the current study uses blood DNA methylation data from the same cohort at 17 years. If the associations with exposure to maternal smoking in cord blood DNA methylation were due to confounding, we would not expect the signal to persist until adolescence. Furthermore, removal of the ALSPAC children from the meta-analysis made no material difference to the effect size estimates (data not shown).

### Sensitivity analyses

#### Impact of offspring's own smoking on their DNA methylation

To assess the impact of participants’ own smoking on methylation level by maternal smoking exposure, the same regression model was run excluding all participants who reported smoking regularly, defined in NFBC1966 and NFBC1986 as smoking at least one cigarette per day for 1 year or more during their life. In the ALSPAC mothers’ cohort, smoking behavior was queried at two time points. At age 30 years, women were asked whether they had smoked regularly before pregnancy. At age 48 years, women were asked whether they were current or former smokers, and in case of the latter, whether they had smoked every day. From these data, a dichotomous variable for any smoking for each of the time points was derived. In the IOWBC, participant’s own smoking status was defined as having ever or never smoked asked via a questionnaire administered at age 18 years. The model was run independently in each study with the same covariates as above (excluding adjustment for offspring’s smoking as all individuals were non-smokers) and meta-analyzed using an inverse-variance weighted fixed-effects model.

#### Impact of a mother’s smoking intensity on offspring DNA methylation

Further analyses were performed to investigate whether the intensity of maternal smoking during pregnancy had a differential impact on the level of offspring blood DNA methylation. For this, the association between the number of cigarettes smoked per day during pregnancy and offspring blood DNA methylation was assessed in the NFBC studies. The association with the number of cigarettes smoked and offspring blood DNA methylation was assessed using linear regression with the same covariates as in the main analysis and meta-analyzed using an inverse variance weighted fixed-effects model.

#### Negative control design to distinguish intrauterine effects from confounding

Potential unmeasured confounding was examined in the NFBC studies by using paternal smoking status during pregnancy as a negative control. This method compares the associations of maternal and paternal smoking during pregnancy with offspring methylation outcomes. Use of paternal smoking as a negative control is based on the assumption that the biological effects of paternal smoking on intrauterine exposure are negligible compared to the effects of maternal smoking during pregnancy. If there is an intrauterine effect of cigarette smoke exposure, the associations are expected to be stronger for maternal smoking than paternal smoking behavior. If effects are of similar magnitude, the associations between maternal smoking during pregnancy and offspring methylation are likely attributable to unmeasured confounding, either by shared environmental or genetic factors [47]. The association with exposure to paternal smoking and offspring blood DNA methylation was assessed using linear regression with the same covariates as in the main analysis and meta-analyzed using an inverse-variance weighted fixed-effects model.

#### Persistence of DNA methylation into adulthood

We also examined whether the methylation changes associated with maternal smoking persisted into middle age. DNA methylation data were available at two time points in NFBC 1966 (age 31 years and 46 years) and ALSPAC mother (age 30 years and 48 years). Generalized least squares were used to examine the longitudinal change in association between exposure to maternal smoking and blood DNA methylation. DNA methylation at each time point was regressed on the technical and white blood cell covariates, and the corresponding residuals were used as the outcome. Study-specific covariates (offspring sex, smoking, BMI, and social class at each time point in NFBC1966; maternal age, social class, and offspring age and smoking status at each time point in ALSPAC) were added in the model. Time point of measurement and its interaction with the exposure were added as additional terms to the regression model, and the model residuals were allowed to be correlated within each individual and be heteroskedastic between time points. The effect estimates at both time points can be derived from this model, and the test for equality of the estimates at both time points is equivalent to testing the interaction term being equal to zero [48]. The analyses were conducted separately in NFBC1966 and ALSPAC mothers and meta-analyzed using an inverse-variance weighted fixed-effects model.

#### Mendelian randomization analysis for the effect of DNA methylation on disease outcomes

We next sought to assess the potential causal relationship between DNA methylation as the exposure and 106 different diseases as outcomes available through the MR-Base platform (available at http://www.mrbase.org/) using two-sample Mendelian randomization (MR). The two-sample MR approach uses gene-exposure and gene-outcome associations from different data sources of comparable populations and allows the interrogation of summary estimates available from large genome-wide association study (GWAS) consortia [24]. If instrumental variable assumptions for the genes associated with the exposure are fulfilled [49], then MR estimates can give evidence for a causal effect of exposure on the outcome.

We first looked up proxy single nucleotide polymorphisms (SNPs) for each of the 69 top maternal smoking-associated CpG sites in the publicly available ARIES database containing methylation quantitative trait loci (mQTL) at four different life stages (birth, childhood, adolescence, middle age) in human blood [42]. We selected SNPs associated with each CpG at *P* < 10^−7^ at any of the other four time points. After clumping SNPs (using 1-Mb window and *R*^2^ < 0.001) and pruning the CpG sites to one per locus, we found strong instruments for 15 CpG sites (Additional file 6). These SNP-CpG associations were consistent across all time points (Additional file 7), except rs4306016-cg01825213 association, which was excluded from the final MR analysis. We selected the SNP-CpG and SNP-disease effect sizes at middle age and aligned these to the same allele. MR effect estimates were then calculated using Wald ratio or, in case of cg04598670, which had two SNP instruments available, inverse-variance weighted method. The resulting effect estimate represents the change in outcome per unit increase in the exposure.

#### Mediation analysis

**Fig. 6:**
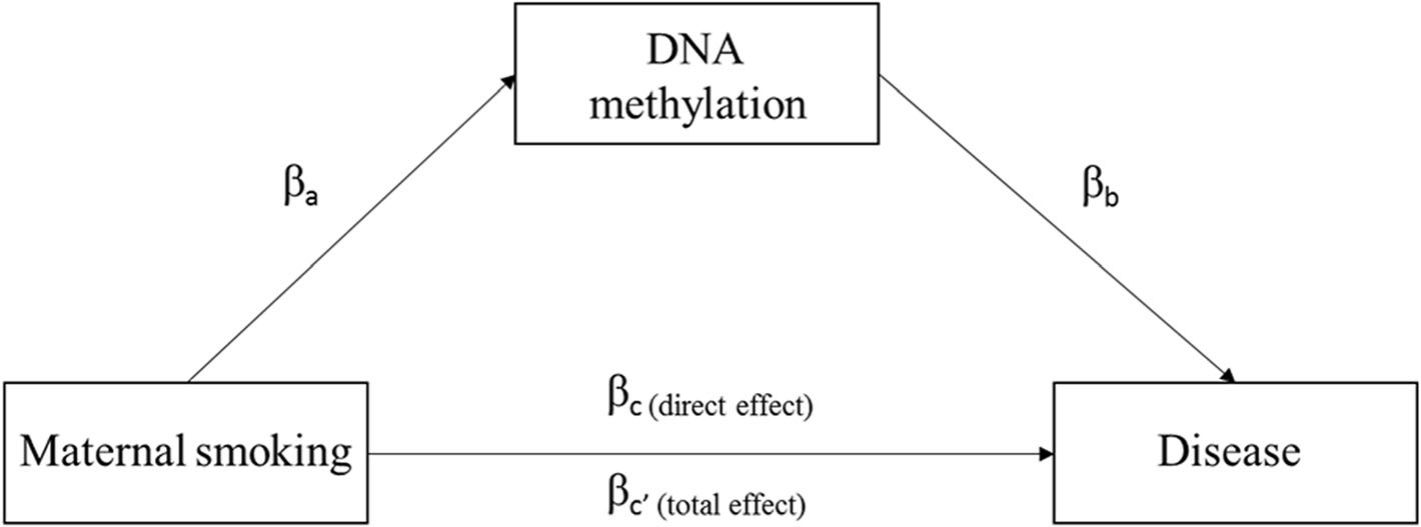
A mediation model for the association between maternal smoking and offspring disease outcomes. *β*_a_ represents the effect estimate for smoking on DNA methylation (CpG = maternal smoking + covariates); *β*_b_ represents the effect estimate for CpG on disease (disease = CpG + covariates); *β*_c_ represents the direct effect (no mediation) estimate for maternal smoking on disease (disease = maternal smoking + covariates; *β*_c′_ represents the total effect estimate on disease (disease = maternal smoking + covariates + CpG)

The CpGs that showed evidence for causal relationship with disease outcomes in the MR analysis were tested for mediation in the association between maternal smoking during pregnancy and disease outcomes using the NFBC1966 data at 31 years and 46 years. We performed model-based causal mediation analysis using R package “mediation” [50] by first estimating both the effect of maternal smoking on the CpG site and the effect of CpG site on the outcome, adjusted for exposure to maternal smoking (Fig. 6). Both of these effects were additionally adjusted for sex, offspring’s own smoking, and technical covariates. We generated the estimates for the total effect, average direct effect, and average causal mediation effect using quasi-Bayesian Monte Carlo method based on normal approximation with 2000 simulations, with robust standard errors. The proportion that the mediating CpG explains of the association between maternal smoking and disease outcome was calculated as described [51].

## Additional files

Additional file 1: Characteristics of the participants based on exposure to maternal smoking during pregnancy in the NFBC cohorts. (DOCX 14 kb)

Additional file 2: Characteristics of the participants based on exposure to maternal smoking during pregnancy in the ALSPAC studies. (DOCX 13 kb)

Additional file 3: Characteristics of the participants based on exposure to maternal smoking during pregnancy in the IOWBC. (DOCX 12 kb)

Additional file 4: Paternal smoking-adjusted association results of exposure to maternal smoking during pregnancy and offspring peripheral blood DNA methylation for the top CpG sites. (DOCX 14 kb)

Additional file 5: Mediation analysis examining the mediated effect of maternal smoking during pregnancy on schizophrenia-related personality traits and inflammatory bowel syndrome in the NFBC 1966 cohort. (DOCX 15 kb)

Additional file 6: CpG sites and their association with methylation in the ARIES cord blood data. (DOCX 14 kb)

Additional file 7: Effect sizes and their 95% confidence intervals of each available SNP-CpG association across different time points in the ARIES data. (DOCX 108 kb)

## Data Availability

The data that support the findings of this study are available from the corresponding author upon request.
