# Supplementary material for: DNA methylation links prenatal smoking exposure to later life health outcomes in offspring
Source: Clin Epigenetics. 2019 Jul 1;11:97. doi: 10.1186/s13148-019-0683-4 (PMC6604191; doi:10.1186/s13148-019-0683-4)
Supplement: Supplementary file 1 — Characteristics of the participants based on exposure to maternal smoking during pregnancy in the NFBC cohorts. (DOCX 14 kb) [file 13148_2019_683_MOESM1_ESM.docx]

Additional file 1. Characteristics of the participants based on exposure to maternal smoking during pregnancy in the NFBC cohorts.

|  | | NFBC 1966 31y | |  | NFBC 1986 16y | | |
| --- | --- | --- | --- | --- | --- | --- | --- |
|  | | Unexposed  n=622 (87%) | Exposed  n=95 (13%) |  | Unexposed  n= 365 (83%) | Exposed  n=77 (17%) | |
| Cumulative exposure in cigs/day* | | 0.0 (0.0 – 2.4) | |  | 0.0 (0.0 – 4.2) | | |
| Males | | 273 (44) | 41 (43) |  | 165 (45) | | 32 (42) |
| Females | | 349 (56) | 54 (57) |  | 200 (55) | | 45 (58) |
| BMI in kg/m^2^ | | 24.5 (4.0) | 25.0 (4.6) |  | 21.3 (3.4) | | 22.1 (4.1) |
| Current smoker | | 229 (37) | 35 (37) |  | 83 (21) | | 33 (38) |
| Own SES at 31y | |  |  | Parental SES at 16y |  | |  |
|  | Upper white-collar | 160 (26) | 19 (20) | Professionals | 293 (80) | | 48 (62) |
|  | Lower white-collar | 218 (35) | 30 (32) | Skilled workers | 52 (14) | | 29 (38) |
|  | Blue-collar | 149 (24) | 29 (31) | Unskilled workers | 3 (1) | | 0 (0) |
|  | Farmer | 11 (2) | 1 (1) | Farmers | 17 (5) | | 0 (0) |
|  | Others | 79 (13) | 14 (15) |  |  | |  |
| Maternal age in years | | 27.7 (6.5) | 25.3 (6.9) |  | 28.3 (5.3) | | 26.3 (5.3) |
| Pre-pregnancy BMI in kg/m^2^ | | 23.4 (3.3) | 22.2 (3.0) |  | 22.4 (3.3) | | 22.0 (3.0) |
| Parental SES at birth | I (highest) | 32 (5) | 2 (2) | Professionals | 109 (30) | | 11 (14) |
|  | II | 111 (18) | 18 (19) | Skilled workers | 154 (43) | | 34 (45) |
|  | III | 250 (40) | 34 (37) | Unskilled workers | 82 (23) | | 31 (41) |
|  | IV (lowest) | 100 (16) | 23 (25) | Farmers | 13 (4) | | 0 (0) |

Data are given as n (%) for categorical variables and mean (standard deviation) for continuous variables.

*Data given as median (10th percentile – 90th percentile). BMI = body mass index; SES = socio-economic position.
