# Supplementary material for: DNA methylation links prenatal smoking exposure to later life health outcomes in offspring
Source: Clin Epigenetics. 2019 Jul 1;11:97. doi: 10.1186/s13148-019-0683-4 (PMC6604191; doi:10.1186/s13148-019-0683-4)
Supplement: Supplementary file 2 — Characteristics of the participants based on exposure to maternal smoking during pregnancy in the ALSPAC studies. (DOCX 13 kb) [file 13148_2019_683_MOESM2_ESM.docx]

|  |  | ALSPAC children at 17y | | ALSPAC mothers 30y | |
| --- | --- | --- | --- | --- | --- |
|  |  | Unexposed | Exposed | Unexposed | Exposed |
|  |  | n= 588 (88 %) | n= 79 (12 %) | n= 536 (71 %) | n= 216 (29 %) |
| Males |  | 306 (52.0) | 39 (49.4) |  |  |
| Females |  | 282 (48.0) | 40 (50.6) | 536 (100%) | 216 (100%) |
| BMI in kg/m^2^ |  | 21.2 (3.3) | 22.3 (3.4) | 22.7 (3.7) | 22.8 (3.5) |
| Current smoker |  | 58* (9.9) | 19* (24.1) | 41 (19.0) | 86 (16.0) |
| Parental SES at child’s age 17y,  Mother’s SES at 30y | |  |  |  |  |
|  | I Professional | 137 (23.3) | 5 (6.3) | 61 (12.2) | 14 (7.1) |
|  | II Managerial and technical | 255 (43.4) | 34 (43.0) | 191 (38.3) | 71 (35.9) |
|  | III (NM) Skilled non-manual | 146 (24.8) | 15 (19.0) | 197 (39.5) | 88 (44.4) |
|  | III (M) Skilled manual | 31 (5.3) | 17 (21.5) | 17 (3.4) | 11 (5.6) |
|  | IV or V Partly skilled or Unskilled | 19 (3.2) | 8 (10.1) | 33 (6.6) | 14 (7.1) |
|  |  |  |  |  |  |
| Maternal age in years | | 30.4 (4.2) | 28.2 (4.5) | 27.5 (5.5) | 27.6 (6.1) |
| Pre-pregnancy BMI in kg/m^2^ | | 22.8 (3.6) | 23.0 (3.9) | - | - |
| Parental SES at child’s birth | I Professional | 65 (12.1) | 21 (9.7) | - | - |
|  | II Managerial and technical | 171 (31.9) | 52 (24.1) | - | - |
|  | III (NM) Skilled non-manual | 69 (12.9) | 28 (13.0) | - | - |
|  | III (M) Skilled manual | 185 (34.5) | 101 (46.8) | - | - |
|  | IV Partly skilled | 30 (5.6) | 9 (4.2) | - | - |
|  | V Unskilled | 16 (3.0) | 5 (2.3) | - | - |

Additional file 2. Characteristics of the participants based on exposure to maternal smoking during pregnancy in the ALSPAC studies.

Data are given as n (%) for categorical variables and mean (standard deviation) for continuous variables. BMI = body mass index; SES = socio-economic position, *Weekly smoking.
