# Supplementary material for: DNA methylation links prenatal smoking exposure to later life health outcomes in offspring
Source: Clin Epigenetics. 2019 Jul 1;11:97. doi: 10.1186/s13148-019-0683-4 (PMC6604191; doi:10.1186/s13148-019-0683-4)
Supplement: Supplementary file 3 — Characteristics of the participants based on exposure to maternal smoking during pregnancy in the IOWBC. (DOCX 12 kb) [file 13148_2019_683_MOESM3_ESM.docx]

Additional file 3. Characteristics of the participants based on exposure to maternal smoking during pregnancy in the Isle of Wight study (IOWBC).

|  |  | Unexposed  n= 215 (84 %) | Exposed  n= 42 (16 %) |
| --- | --- | --- | --- |
| Males |  | 67 (31.2) | 16 (32.1) |
| Females |  | 148 (68.8) | 26 (61.9) |
| BMI in kg/m^2^ |  | 23.10 (3.8) | 24.37 (5.3) |
| Current smoker |  | 56 (26.1) | 14 (33.3) |
| Own SES at 18y | |  |  |
|  | I LowLow | 29(13.5) | 13 (31.0) |
|  | II Low | 37(17.2) | 9 (21.4) |
|  | III LowMid | 49(22.8) | 19 (45.2) |
|  | IV Mid | 71(33.0) | 1 (2.4) |
|  | V High | 29(13.5) | - |
|  |  |  |  |
| Maternal age in years | | 28.1 (5.4) | 27.0 (5.6) |
| Maternal Pre-pregnancy BMI in kg/m^2^ | | 24.11 (3.6) | 24.24 (4.9) |
| Parental SES | I LowLow | 84 (39.1) | 11 (26.2) |
|  | II Low | 58 (27.0) | 9 (21.4) |
|  | III LowMid | 66 (30.7) | 13 (31.0) |
|  | IV Mid | 7 (3.3) | 9 (21.4) |
|  |  |  |  |
|  |  |  |  |

Data are given as n (%) for categorical variables and mean (standard deviation) for continuous variables.

BMI = body mass index; SES = socio-economic position.
