# Supplementary material for: DNA methylation links prenatal smoking exposure to later life health outcomes in offspring
Source: Clin Epigenetics. 2019 Jul 1;11:97. doi: 10.1186/s13148-019-0683-4 (PMC6604191; doi:10.1186/s13148-019-0683-4)
Supplement: Supplementary file 4 — Paternal smoking-adjusted association results of exposure to maternal smoking during pregnancy and offspring peripheral blood DNA methylation for the top CpG sites. (DOCX 14 kb) [file 13148_2019_683_MOESM4_ESM.docx]

Additional file 4. Paternal smoking adjusted association results of exposure to maternal smoking during pregnancy and offspring peripheral blood DNA Methylation for the top CpG sites.

| Name | Chr | MAPINFO | Gene | B | SE | P |
| --- | --- | --- | --- | --- | --- | --- |
| cg15325070 | 1 | 2792704 |  | 0.015 | 0.003 | 3.20E-06 |
| cg25189904 | 1 | 68299493 | *GNG12* | -0.024 | 0.004 | 4.20E-11 |
| cg14179389 | 1 | 92947961 | *GFI1* | -0.025 | 0.004 | 4.70E-12 |
| cg04749740 | 2 | 65935124 |  | 0.014 | 0.003 | 3.40E-05 |
| cg06635952 | 2 | 70025869 | *ANXA4* | 0.008 | 0.002 | 0.00071 |
| cg11025974 | 2 | 152830521 | *CACNB4* | 0.016 | 0.003 | 1.60E-08 |
| cg14157435 | 2 | 206628692 | *NRP2* | -0.038 | 0.007 | 4.90E-07 |
| cg05783384 | 2 | 218843735 |  | 0.015 | 0.005 | 0.00062 |
| cg05204104 | 2 | 235403141 | *ARL4C* | 0.02 | 0.003 | 1.10E-08 |
| cg00174179 | 3 | 49450293 | *RHOA;TCTA* | -0.006 | 0.001 | 3.20E-05 |
| cg16449012 | 4 | 17781880 | *FAM184B* | 0.013 | 0.003 | 2.90E-05 |
| cg05575921 | 5 | 373378 | *AHRR* | -0.018 | 0.003 | 1.10E-08 |
| cg21253335 | 5 | 87835928 |  | 0.017 | 0.003 | 1.80E-06 |
| cg01952185 | 5 | 134813213 |  | 0.02 | 0.003 | 1.10E-10 |
| cg05634495 | 6 | 122364658 |  | 0.017 | 0.004 | 2.50E-06 |
| cg00794911 | 6 | 166260532 |  | -0.01 | 0.002 | 4.40E-06 |
| cg25879142 | 7 | 4671391 |  | 0.022 | 0.004 | 4.10E-09 |
| cg20117519 | 7 | 8429907 |  | 0.023 | 0.005 | 4.20E-07 |
| cg19089201 | 7 | 45002287 | *MYO1G* | 0.028 | 0.003 | 3.50E-17 |
| cg04598670 | 7 | 68697651 |  | -0.013 | 0.004 | 0.0014 |
| cg25949550 | 7 | 145814306 | *CNTNAP2* | -0.006 | 0.001 | 4.50E-14 |
| cg11207515 | 7 | 146904205 | *CNTNAP2* | -0.018 | 0.004 | 1.40E-06 |
| cg15578140 | 7 | 147718109 | *MIR548F3;CNTNAP2* | 0.012 | 0.002 | 6.60E-07 |
| cg17199018 | 8 | 28206278 | *ZNF395* | -0.012 | 0.003 | 0.00049 |
| cg14563637 | 9 | 98931801 |  | 0.016 | 0.003 | 6.20E-08 |
| cg14540913 | 9 | 132458514 | *PRRX2* | 0.015 | 0.003 | 2.90E-08 |
| cg13822849 | 9 | 137999757 | *OLFM1* | 0.006 | 0.002 | 0.0003 |
| cg11813497 | 10 | 14372879 | *FRMD4A* | 0.025 | 0.004 | 2.40E-12 |
| cg05697249 | 11 | 111789693 | *C11orf52* | 0.014 | 0.003 | 4.50E-08 |
| cg18493761 | 11 | 125386885 |  | 0.034 | 0.005 | 7.80E-13 |
| cg05549655 | 15 | 75019143 | *CYP1A1* | 0.01 | 0.001 | 1.40E-17 |
| cg13834112 | 15 | 90361639 |  | 0.016 | 0.003 | 2.20E-08 |
| cg00253658 | 16 | 54210496 |  | 0.039 | 0.005 | 2.30E-13 |
| cg04358214 | 16 | 67143304 | *C16orf70* | 0.019 | 0.005 | 0.00021 |
| cg12984635 | 19 | 44032076 | *ETHE1* | 0.018 | 0.003 | 1.50E-10 |
| cg06758350 | 21 | 36259460 | *RUNX1* | 0.027 | 0.006 | 3.70E-06 |

Chr = chromosome; B = effect size estimate, SE = standard error.
