# Supplementary material for: DNA methylation links prenatal smoking exposure to later life health outcomes in offspring
Source: Clin Epigenetics. 2019 Jul 1;11:97. doi: 10.1186/s13148-019-0683-4 (PMC6604191; doi:10.1186/s13148-019-0683-4)
Supplement: Supplementary file 5 — Mediation analysis examining the mediated effect of maternal smoking during pregnancy on schizophrenia-related personality traits and inflammatory bowel syndrome in the NFBC 1966 cohort. (DOCX 15 kb) [file 13148_2019_683_MOESM5_ESM.docx]

Additional file 5. Mediation analysis examining the mediated effect of maternal smoking during pregnancy on schizophrenia-related personality traits and inflammatory bowel syndrome in the NFBC 1966 cohort.

| **Effect on BIP2 via cg25189904** |  |  |  |  |
| --- | --- | --- | --- | --- |
|  | β | CIL | CIU | *P* value |
| ACME | 0.07 | 0.01 | 0.14 | 0.024 |
| ADE | 0.16 | -0.05 | 0.36 | 0.15 |
| Total Effect | 0.22 | 0.03 | 0.43 | 0.026 |
| Estimated proportion mediated | 0.30 |  |  |  |
|  |  |  |  |  |
| **Effect on HPS via cg25189904** |  |  |  |  |
|  | β | CIL | CIU | *P* value |
| ACME | 0.07 | 0.01 | 0.14 | 0.018 |
| ADE | 0.16 | -0.08 | 0.40 | 0.19 |
| Total Effect | 0.23 | 0.00 | 0.47 | 0.05 |
| Estimated proportion mediated | 0.28 |  |  |  |
|  |  |  |  |  |
| **Effect on PER via cg25189904** |  |  |  |  |
|  | β | CIL | CIU | *P* value |
| ACME | 0.02 | -0.04 | 0.07 | 0.57 |
| ADE | 0.25 | -0.05 | 0.55 | 0.12 |
| Total Effect | 0.26 | -0.04 | 0.57 | 0.092 |
| Estimated proportion mediated | 0.05 |  |  |  |
|  |  |  |  |  |
| **Effect on PAS via cg25189904** |  |  |  |  |
|  | β | CIL | CIU | *P* value |
| ACME | 0.04 | -0.01 | 0.10 | 0.13 |
| ADE | 0.11 | -0.15 | 0.35 | 0.37 |
| Total Effect | 0.15 | -0.10 | 0.37 | 0.21 |
| Estimated proportion mediated | 0.19 |  |  |  |
|  |  |  |  |  |
| **Effect on SAS via cg25189904** |  |  |  |  |
|  | β | CIL | CIU | *P* value |
| ACME | 0.02 | -0.04 | 0.07 | 0.55 |
| ADE | 0.21 | -0.06 | 0.48 | 0.12 |
| Total Effect | 0.23 | -0.03 | 0.49 | 0.079 |
| Estimated proportion mediated | 0.05 |  |  |  |
|  |  |  |  |  |
| **Effect on SCH via cg25189904** |  |  |  |  |
|  | β | CIL | CIU | *P* value |
| ACME | -0.01 | -0.08 | 0.06 | 0.86 |
| ADE | 0.08 | -0.17 | 0.34 | 0.52 |
| Total Effect | 0.08 | -0.16 | 0.33 | 0.55 |
| Estimated proportion mediated | 0 |  |  |  |
|  |  |  |  |  |
| **Effect on inflammatory bowel syndrome via cg15578140** | | | | |
|  | β | CIL | CIU | *P* value |
| ACME | 0.00 | -0.02 | 0.03 | 0.83 |
| ADE | 0.08 | -0.03 | 0.21 | 0.15 |
| Total Effect | 0.09 | -0.02 | 0.21 | 0.13 |
| Estimated proportion mediated | 0.02 |  |  |  |
|  |  |  |  |  |
| **Effect on inflammatory bowel syndrome via cg09935388** | | | | |
|  | β | CIL | CIU | *P* value |
| ACME | 0.01 | -0.01 | 0.03 | 0.39 |
| ADE | 0.08 | -0.03 | 0.21 | 0.18 |
| Total Effect | 0.08 | -0.02 | 0.22 | 0.14 |
| Estimated proportion mediated | 0.06 |  |  |  |
|  |  |  |  |  |
| **Effect on inflammatory bowel syndrome via cg04598670** | | | |  |
|  | β | CIL | CIU | *P* value |
| ACME | -0.01 | -0.03 | 0.01 | 0.36 |
| ADE | 0.09 | -0.02 | 0.23 | 0.097 |
| Total Effect | 0.09 | -0.02 | 0.22 | 0.13 |
| Estimated proportion mediated | 0 |  |  |  |

ACME = Average Causal Mediated Effect; ADE = Average Direct Effect, β = effect size;

CIL = 95 % Confidence Interval, Lower limit; CIU = 95 % Confidence Interval, Upper limit; BIP2 = Bipolar II Scale; HPS = Hypomanic Personality Scale; PER = Perceptual Aberration Scale; PAS = Physical Anhedonia Scale; SAS = Social Anhedonia Scale; SCH = Schizoidia Scale.
