# Supplementary material for: DNA methylation links prenatal smoking exposure to later life health outcomes in offspring
Source: Clin Epigenetics. 2019 Jul 1;11:97. doi: 10.1186/s13148-019-0683-4 (PMC6604191; doi:10.1186/s13148-019-0683-4)
Supplement: Supplementary file 6 — CpG sites and their association with methylation in the ARIES cord blood data. (DOCX 14 kb) [file 13148_2019_683_MOESM6_ESM.docx]

Additional file 6. CpG site and their association with methylation in the ARIES cord blood data.

| CpG | SNP | Chr | Position | Gene | EA | NEA | EAF | B (SE) |  |
| --- | --- | --- | --- | --- | --- | --- | --- | --- | --- |
| cg01825213 | rs4306016 | 9 | 98987400 | *HSD17B3* | T | C | 0.5 | -0.28 (0.03) | cis |
| cg03142697 | rs11088296 | 21 | 36237545 | *RUNX1* | T | C | 0.26 | 0.22 (0.03) | cis |
| cg04598670 | rs12667374 | 7 | 68701971 | *LOC100507468* | A | G | 0.06 | 0.49 (0.06) | cis |
| cg04598670 | rs115340020 | 1 | 92766437 | *RPAP2* | A | G | 0.02 | -0.37 (0.10) | trans |
| cg05204104 | rs1464264 | 2 | 235199586 | *ARL4C* | G | A | 0.39 | -0.11 (0.02) | cis |
| cg05348875 | rs79024572 | 2 | 205944964 | *PARD3B* | T | G | 0.05 | -1.10 (0.12) | cis |
| cg09935388 | rs115340020 | 1 | 92766437 | *RPAP2* | A | G | 0.02 | -1.01 (0.19) | cis |
| cg11429111 | rs7701950 | 5 | 134789600 | *TIFAB* | C | G | 0.15 | 0.09 (0.03) | cis |
| cg12803068 | rs61087358 | 7 | 45017742 | *MYO1G* | A | G | 0.2 | -0.62 (0.08) | cis |
| cg15578140 | rs4725759 | 7 | 147715701 | *MIR548T;CNTNAP2;MIR548F3* | G | T | 0.29 | -0.14 (0.03) | cis |
| cg17924476 | rs13167067 | 5 | 195506 | *LRRC14B* | C | T | 0.41 | 0.26 (0.03) | cis |
| cg18493761 | rs588399 | 11 | 125348354 | *FEZ1* | G | T | 0.22 | -0.23 (0.04) | cis |
| cg21253335 | rs11750802 | 5 | 87795826 | *LINC00461* | G | A | 0.14 | -0.30 (0.04) | cis |
| cg25189904 | rs7540541 | 1 | 68299551 | *GNG12-AS1* | G | T | 0.38 | 0.20 (0.03) | cis |
| cg25879142 | rs8046907 | 16 | 67591388 | *CTCF* | G | A | 0.13 | 0.31 (0.05) | trans |

SNP = Single nucleotide polymorphisms; Chr = chromosome; EA = effect allele; NEA = non-effect allele; EAF = effect allele frequency; B = effect size estimate, SE = standard error.
