# Supplementary material for: DNA methylation links prenatal smoking exposure to later life health outcomes in offspring
Source: Clin Epigenetics. 2019 Jul 1;11:97. doi: 10.1186/s13148-019-0683-4 (PMC6604191; doi:10.1186/s13148-019-0683-4)
Supplement: Supplementary file 7 — Effect sizes and their 95% confidence intervals of each available SNP-CpG association across different time points in the ARIES data. (DOCX 108 kb) [file 13148_2019_683_MOESM7_ESM.docx]

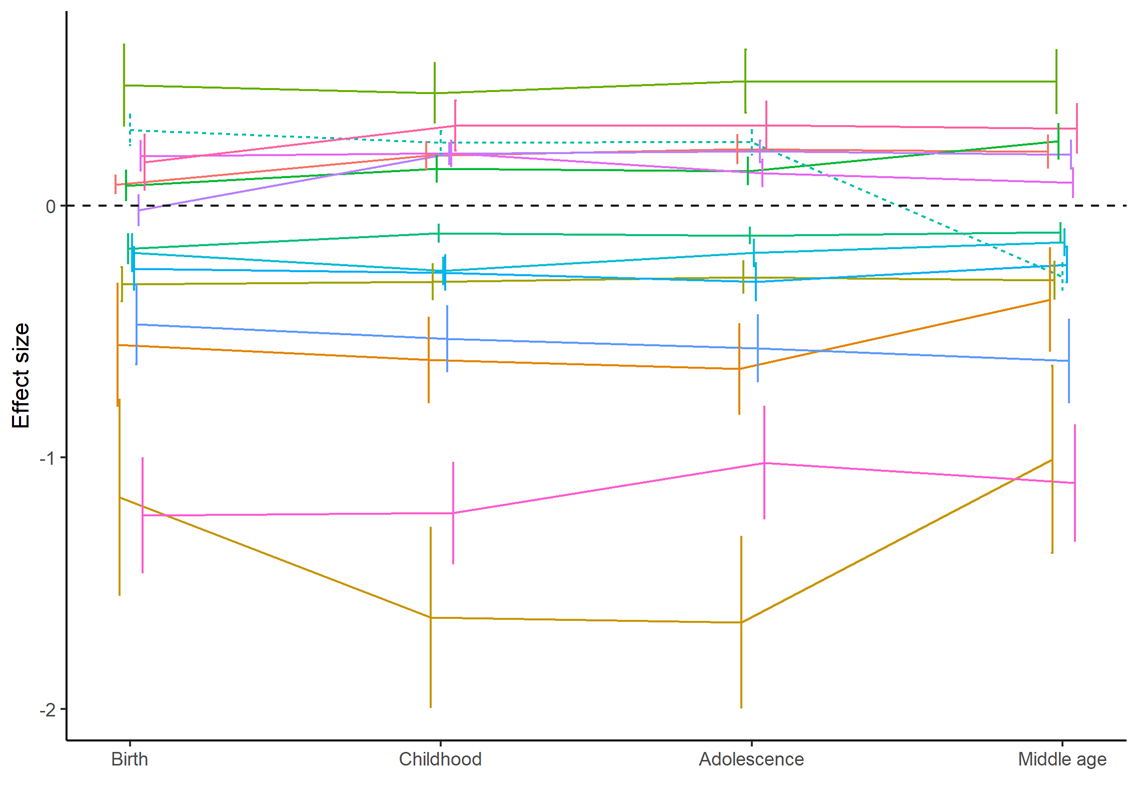


Additional file 7. Effect sizes and their 95% confidence intervals of each available SNP-CpG association across different time point in the ARIES data. Horizontal lines represent the same SNP-CpG association at each time point. Dotted green line indicates SNP-CpG association that was not consistent across all time points.
